# Optimisation of the Flame Spheroidisation Process for the Rapid Manufacture of Fe_3_O_4_-Based Porous and Dense Microspheres

**DOI:** 10.3390/molecules28062523

**Published:** 2023-03-09

**Authors:** Jesús Molinar-Díaz, John Luke Woodliffe, Elisabeth Steer, Nicola A. Morley, Paul D. Brown, Ifty Ahmed

**Affiliations:** 1 Advanced Materials Research Group, Faculty of Engineering, University of Nottingham, University Park, Nottingham NG7 2RD, UK; 2 Nanoscale and Microscale Research Centre, University of Nottingham, University Park, Nottingham NG7 2RD, UK; 3 Department of Materials Science and Engineering, University of Sheffield, Sheffield S1 3JD, UK

**Keywords:** magnetite, magnetic particles, porous microspheres, calcium ferrites, flame spheroidisation, ceramics, magnetic hyperthermia

## Abstract

The rapid, single-stage, flame-spheroidisation process, as applied to varying Fe_3_O_4_:CaCO_3_ powder combinations, provides for the rapid production of a mixture of dense and porous ferromagnetic microspheres with homogeneous composition, high levels of interconnected porosity and microsphere size control. This study describes the production of dense (35–80 µm) and highly porous (125–180 µm) Ca_2_Fe_2_O_5_ ferromagnetic microspheres. Correlated backscattered electron imaging and mineral liberation analysis investigations provide insight into the microsphere formation mechanisms, as a function of Fe_3_O_4_/porogen mass ratios and gas flow settings. Optimised conditions for the processing of highly homogeneous Ca_2_Fe_2_O_5_ porous and dense microspheres are identified. Induction heating studies of the materials produced delivered a controlled temperature increase to 43.7 °C, indicating that these flame-spheroidised Ca_2_Fe_2_O_5_ ferromagnetic microspheres could be highly promising candidates for magnetic induced hyperthermia and other biomedical applications.

## 1. Introduction

From the magnetic materials available, Fe_3_O_4_-based superparamagnetic nanoparticles (SMNPs) have been most extensively investigated for localised magnetic hyperthermia applications due to their superparamagnetic expression and non-toxicity [1,2], and because iron oxide metabolism is readily achieved by the heme oxygenase-1 gene which generates haemoglobin and promotes cellular iron homeostasis [3]. Related ferrites, such as NiFe_2_O_4_ [4], MnFe_2_O_4_ [5], CoFe_2_O_4_ [6] and Li_x_Fe_3-x_O_4_ [7], have also been investigated to improve magnetic strength and thermal stability. However, the inherent toxicity of Ni, Mn, Co and Li limits their application [3]. An alternative approach is to introduce non-magnetic Ca^2+^ into the ferrite crystalline structure, to generate significant improvements in terms of biocompatibility, whilst maintaining magnetic expression and heating control [8,9]. Non-toxic calcium ferrites have been shown to metabolise safely within the body [10,11], making them appropriate for a range of biomedical applications, including magnetic hyperthermia. Biomedical investigations using calcium ferrites have been reported in relation to drug-delivery systems [8,9,12,13] and cytocompatibility [8,10,12,14]. Approaches combining metal cations and calcium ferrites for therapeutic applications have also been explored [9,11,15,16,17,18]. The present challenge is to develop these materials into practical morphologies for enhanced biomedical investigations.

Microspheres have been widely investigated for a variety of healthcare applications. Porous microspheres, in particular, offer functional advantages, including the incorporation of payloads (drugs, cells, biologics, etc.) on external surfaces and within their pores [19,20], along with cell attachment and proliferation over the enhanced surface area available [19]. However, biomedical applications of microspheres are dependent strongly on the types of material employed. In the case of polymer-based microspheres, applications have included targeted drug delivery, tissue engineering and regenerative medicine [21,22]. Whilst ceramic and glass-based microspheres are more commonly associated with tissue regeneration applications, e.g., bone repair [23,24], orthopaedics [25], dental [26] and cancer therapeutics (radiotherapy) [27], along with magnetic hyperthermia.

Raising the temperature of cancerous regions of the body to 40–45 °C to induce cell death is the basis for hyperthermia treatment [3,28,29], whereas magnetic hyperthermia treatment utilises an alternating magnetic field (AMF) to produce localised heat using magnetic particles [30]. Most research on magnetic hyperthermia has focused on SMNPs, which show remarkable physical and functional properties, useful for magnetic hyperthermia, including remote manipulation mediated by an AMF [31], the ability to cross biological barriers due to their small size (10–100 nm) [32], biocompatibility [33] and capability to transform AMF energy into heat [31]. A limitation of SMNPs’ use on local magnetic hyperthermia relates to the insufficient amount of heat generated by a single nanoparticle [34,35], with the agglomeration of large quantities of SMNPs required to produce sufficient heat to trigger cancer cell death [36,37]. However, agglomeration can have a negative effect on superparamagnetic properties and heat performance of SMNPs [36,38]. Alternatively, it has been suggested to use larger (≥1 µm) ferromagnetic particles [39].

Ceramic and glass-ceramic microspheres can be manufactured by a variety of methods, including vertical furnace drop tower [19], sol-gel [40], spray drying [41] and flame spheroidisation approaches [42]. In particular, the single-stage, flame spheroidisation process offers a rapid, cost-effective [19,25] and promising approach for the large-scale manufacture of porous and dense microspheres. The feeding of fine-scale particles into a high-temperature flame causes them to melt and acquire spherical form upon ejection from the flame as a consequence of surface tension and solidification upon cooling [42,43]. A variety of physical and process parameters associated with flame spheroidisation can affect the morphology of the end microsphere products [43], including feedstock particle size, material melting temperature, flame temperature and gas flow ratio.

In this context, we report on the flame spheroidisation-processing of Fe_3_O_4_:CaCO_3_ powders, and the optimisation of mass ratio and O_2_/C_2_H_2_ gas flow conditions for the production of porous and dense, calcium ferrite microspheres, with highly controlled microsphere sizes and morphologies. Complementary magnetisation and induction heating investigations have also been undertaken to validate the potential of Ca_2_Fe_2_O_5_ microspheres for magnetic hyperthermia applications.

## 2. Results

### 2.1. Effect of Fe_3_O_4_:CaCO_3_ Mass Ratio 

#### 2.1.1. Size Range of Microsphere Reaction Products (Unsieved)

**Figure 1:**
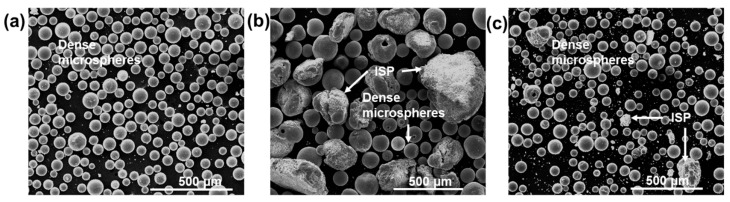
SE images of flame-spheroidised Fe_3_O_4_:CaCO_3_, using starting ≤ 5 µm sized Fe_3_O_4_ powders. Mass ratios: (**a**) 3:1, (**b**) 1:1 and (**c**) 1:3, (unsieved), showing dense microspheres and irregular-shaped particles. ISP: irregular-shaped particles.

**Figure 2:**
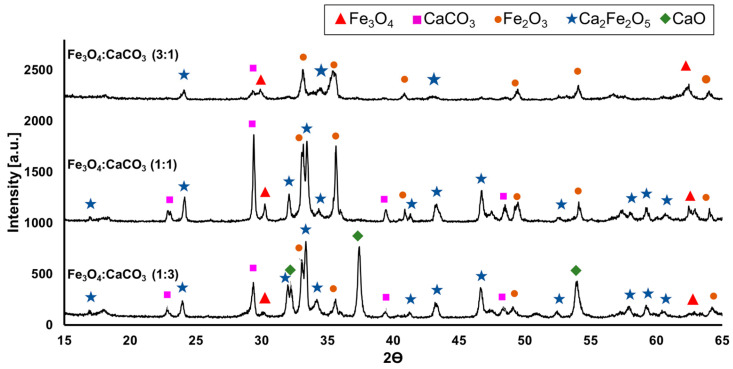
XRD patterns for flame-spheroidised Fe_3_O_4_:CaCO_3_ (mass ratios 3:1, 1:1 and 1:3).

**Figure 3:**
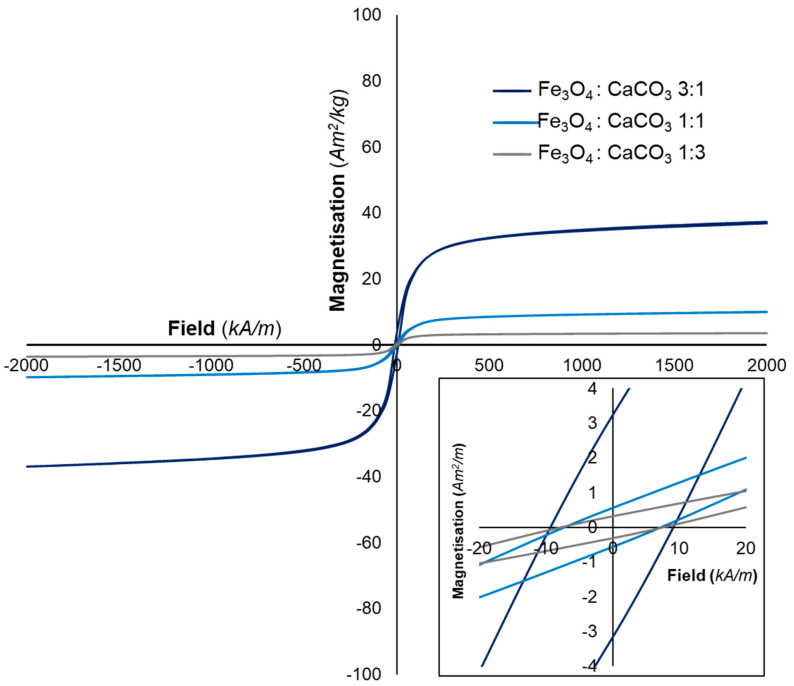
Magnetisation curves for flame-spheroidised Fe_3_O_4_:CaCO_3_ (mass ratios 3:1, 1:1 and 1:3; GFS 2.5:2.5) at 26.85 °C. Inset figure provides evidence of remanent magnetisation in all cases.

Figure 1a–c shows low magnification SE images of flame-spheroidised products formed from Fe_3_O_4_:CaCO_3_ precursor/porogen materials, with mass ratios of Figure 1a—3:1, Figure 1b—1:1 and Figure 1c—1:3, respectively, using a gas flow setting of 2.5:2.5 in all cases.

As summarised in Table 1, a mass ratio of 1:1 led to the production of a mixture of dense microspheres and larger, irregular-shaped particles (Figure 1b). Notably, a 3:1 mass ratio (Fe_3_O_4_-rich) generated a consistent yield of dense microspheres with very few irregular-shaped particles (Figure 1a), whereas a 1:3 mass ratio (CaCO_3_-rich) produced a similar mixture of dense microspheres and few irregular-shaped particles (Figure 1c).

#### 2.1.2. Structural Characterisation (Unsieved Microspheres)

XRD investigations were performed to appraise the structural integrity of unsieved reaction products as a function of the flame-spheroidisation processing conditions. Figure 2 shows XRD patterns for the flame-spheroidised products (mass ratios 3:1, 1:1 and 1:3) corresponding to the sample sets imaged in Figure 1a–c. Structural analyses confirmed the presence of varying proportions of Ca_2_Fe_2_O_5_ (srebrodolskite) (ICDD PDF no. 00-047-1744), Fe_3_O_4_ (magnetite) (ICDD PDF no. 01-087-0244), Fe_2_O_3_ (hematite) (ICDD PDF 00-033-0664), CaCO_3_ (calcium carbonate) (ICDD PDF no. 00-047-1743) and CaO (calcium oxide) (ICDD PDF no. 00-048-1467).

As outlined in Table S1 (see Supplementary Materials), a mass ratio of 1:1 produced strong signatures for Fe_2_O_3_, Ca_2_Fe_2_O_5_ and (unreacted) CaCO_3_, along with a medium signature for (unreacted) Fe_3_O_4_. A mass ratio of 3:1 (Fe_3_O_4_-rich) revealed a dominant Fe_2_O_3_ signature, with medium Fe_3_O_4_ and Ca_2_Fe_2_O_5_ signatures, and a weak signature for CaCO_3_. A mass ratio of 1:3 (CaCO_3_-rich) showed strong signatures for Ca_2_Fe_2_O_5_ and CaO, medium signatures for Fe_2_O_3_ and CaCO_3_, and a weak signature for Fe_3_O_4_. In particular, the progression towards higher porogen content (mass ratio from 3:1 to 1:3) was associated with a reduction in magnetite (Fe_3_O_4_) and hematite (Fe_2_O_3_) peak intensities and a consolidation of intensities attributable to srebrodolskite (Ca_2_Fe_2_O_5_), CaCO_3_ and CaO (reacted porogen).

#### 2.1.3. Microsphere Magnetic Properties (Sieved)

Magnetisation measurements provided information on the magnetic properties of flame spheroidised microspheres and clarified the effect of Fe_3_O_4_:CaCO_3_ mass ratio on magnetic expression. For all samples, the magnetisation curves were indicative of typical ferrimagnetic behaviour. Figure 3 presents magnetisation curves for the sieved flame-spheroidised products (mass ratios 3:1, 1:1 and 1:3; gas flow setting 2.5:2.5). As summarised in Table 2, progression towards increased porogen (mass ratio from 3:1 to 1:3) was accompanied by a decrease in magnetisation, consistent with a lowering of iron content.

#### 2.1.4. Fine Scale Morphologies and Compositional Analyses (Sieved and Sectioned)

**Figure 4:**
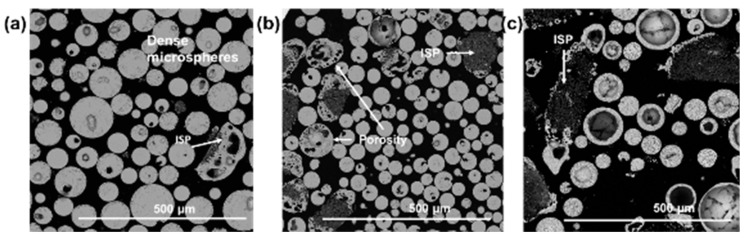

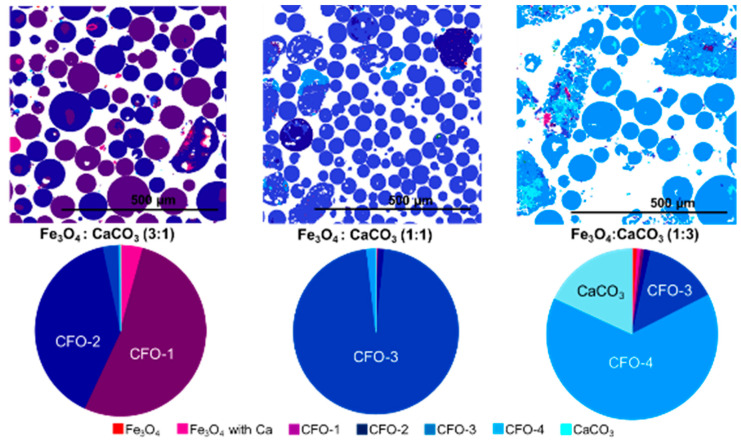
BSE images, MLA compositional analyses and associated pie-charts showing mineral proportions (wt%) of flame spheroidised Fe_3_O_4_:CaCO_3_ as a function of mass ratio: (**a**) 3:1, (**b**) 1:1 and (**c**) 1:3; following sieving and sectioning, illustrating microsphere porosity, ISP, and dense microspheres. ISP: irregular-shaped particles; CFO: calcium iron oxide.

Figure 4a–c presents BSE images and MLA mapping analyses, revealing fine-scale morphological details and clarifying the outcomes of compositional dependencies on the precursor-porogen mass ratio of the sieved microsphere products, extracted from the sample sets presented in Figure 1. 

Varying levels of internal porosity were observed for all sample sets. For the case of flame-spheroidised Fe_3_O_4_:CaCO_3_ with mass ratio 1:1/gas flow setting 2.5:2.5 (Figure 4b), upon sectioning, direct evidence was provided for the development of low and high levels of internal porosity within dense microspheres and larger irregular-shaped particles, respectively. For the case of flame-spheroidised processed precursor-rich powder (mass ratio 3:1; gas flow setting 2.5:2.5; Figure 4a), the evidence showed the development of comparatively lower levels of internal porosity within dense microspheres and few irregular-shaped particles. Conversely, for the case of flame-spheroidised processed porogen-rich powder (mass ratio 1:3; gas flow setting 2.5:2.5; Figure 4c), a variety of dense and irregular-shaped developed morphologies with significantly higher levels of internal porosity was revealed. 

In particular, and as highlighted in Table 3, compositional differences were evident across the mass ratio sample set, with a strong trend towards the development of banded calcium iron oxide (CFO) compositions, the most prevalent of which being srebrodolskite (Ca_2_Fe_2_O_5_, denoted CFO-3—Ca_2_Fe_2_O_5_; Supplementary Materials, Table S2 and Figure S1). As anticipated, a progressive decrease in precursor content or increment in porogen content (mass ratio 3:1 to 1:3) was directly accompanied by a lowering of Fe levels and elevation of Ca levels throughout the microsphere products.

For the case of flame spheroidised Fe_3_O_4_:CaCO_3_ with a mass ratio of 1:1 gas flow setting 2.5:2.5 (Figure 4b; Supplementary Materials, Figure S2), MLA mapping provided direct evidence for high levels of homogeneity of CFO-3—Ca_2_Fe_2_O_5_. For the case of flame-spheroidised precursor-rich powder (mass ratio 3:1; gas flow setting 2.5:2.5; Figure 4a, Supplementary Materials, Figure S3), the evidence comprised two dominant CFO levels (denoted CFO-1 and CFO-2; Supplementary Materials, Table S2). Both of these samples (Figure 4a,b) showed complete consumption of the precursor and porogen, with no evidence of residual Fe_3_O_4_ nor CaCO_3_. Conversely, for the case of flame spheroidised porogen-rich powder with a mass ratio of 1:3 (Figure 4c, Supplementary Materials, Figure S4), the data revealed a mixture of CFO compositions dominated by Ca excess (CFO-4; Supplementary Materials, Table S2), along with larger irregular-shaped particles comprising unreacted porogen.

Complementary, energy-dispersive X-ray spectroscopy (EDS) mappings validated elemental compositions and fine-scale details for the microsphere products, as a function of mass ratios of 3:1, 1:1 and 1:3 (Supplementary Materials, Figure S5 and Table S3).

### 2.2. Effect of O_2_/C_2_H_2_ Gas Flow Setting

#### 2.2.1. Size Range of Microsphere Reaction Products (Unsieved)

**Figure 5:**
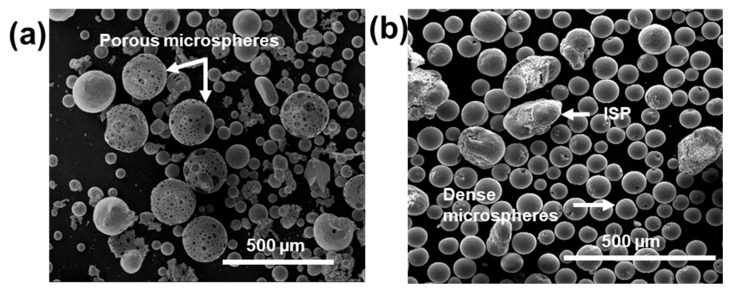
SE images of flame-spheroidised Fe_3_O_4_:CaCO_3_ (1:1 mass ratio). Gas flow settings: (**a**) 2:2 and (**b**) 3:3 (unsieved). ISP: irregular-shaped particles.

Figure 5a,b shows low magnification SE images of flame-spheroidised Fe_3_O_4_:CaCO_3_ products, for precursor to porogen (Fe_3_O_4_:CaCO_3_) mass ratio 1:1, with gas flow settings of Figure 5a—2:2 and Figure 5b—3:3, respectively.

As summarised in Table 4, a lower gas flow setting of 2:2 (mass ratio 1:1) resulted in a consistent yield of highly porous microspheres, along with a small dense microsphere and very few irregular-shaped particles (Figure 5a). Comparatively, an increased gas flow setting of 3:3 (mass ratio 1:1) resulted in dense microspheres and few irregular-shaped particles (Figure 5b). Notably, only the gas flow setting 2:2 (mass ratio 1:1) processing conditions resulted in the production of microspheres with visible evidence for porosity (pore size range 1.8–64.5 µm; mean pore size 13.1 µm, SD 12.6 µm; ImageJ software).

#### 2.2.2. Fine Scale Morphologies and Compositional Analyses (Sieved and Sectioned)

**Figure 6:**
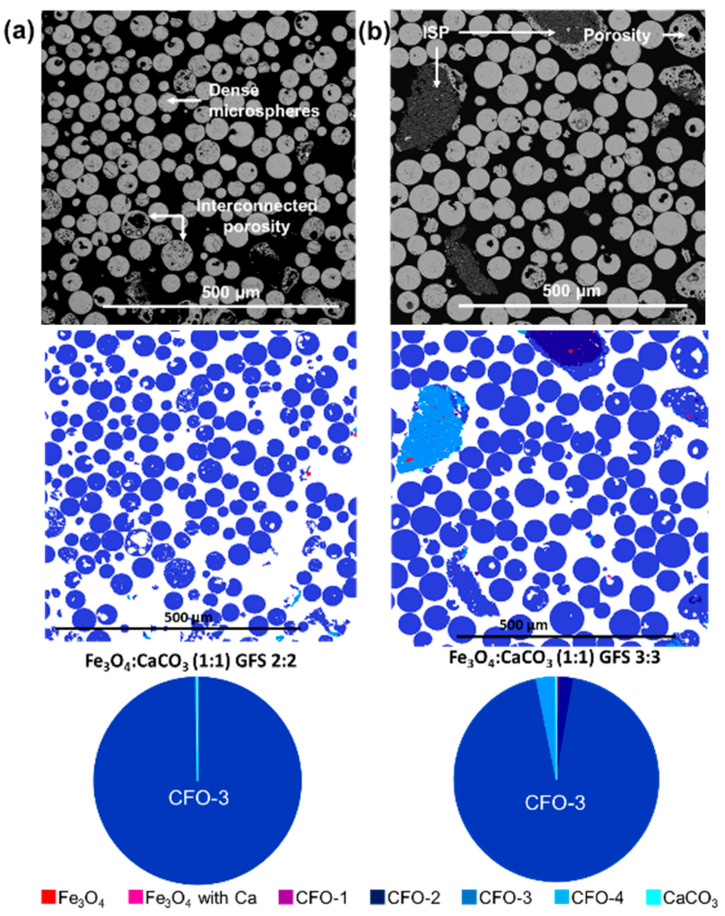
BSE images, MLA compositional analysis and associated pie-charts showing the mineral proportions (wt%) of flame-spheroidised Fe_3_O_4_:CaCO_3_ (mass ratio 1:1) microspheres: (**a**) 2:2 and (**b**) 3:3, following sieving and sectioning, illustrating microspheres, porosity and minerals obtained. Mineral references shown on Table S2. GFS: gas flow setting.

Figure 6a,b show BSE images and MLA mineral mapping analyses, extracted from the sample sets presented in Figure 5, presenting fine-scale morphological details of the sieved microsphere products and clarifying the compositional dependencies on gas flow setting. Interestingly, significant levels of internal porosity were revealed for both sample sets. For the case of the higher gas flow setting 3:3, moderate levels of internal porosity were associated with the microspheres and a few irregular-shaped products (Figure 6b). Whereas, for gas flow setting 2:2, a variety of developed porosities was evident, including high levels of interconnected porosity for the case of larger microspheres (125–180 µm) (Figure 6a).

As highlighted in Table 5, high levels of sample homogeneity were maintained, as a function of the gas flow setting, with CFO-3 srebrodolskite (Ca_2_Fe_2_O_5_) as the dominant phase for all mass ratio 1:1 sample sets. For the case of gas flow setting 3:3, the Ca_2_Fe_2_O_5_ proportion decreased slightly compared to gas flow setting 2.5:2.5 (Table 3 and Table 5), whilst a small amount of CFO-2 and CFO-4 was evident (Figure 6b; Supplementary Materials, Figure S6). Notably, for the case of gas flow setting 2:2, the highest levels of Ca_2_Fe_2_O_5_ homogeneity (99.6 wt%) were returned (Figure 6a; Supplementary Materials, Figure S7).

It was noted that the flame spheroidised Fe_3_O_4_:CaCO_3_ (1:1 mass ratio; 2:2 gas flow setting) samples revealed the highest levels of compositional uniformity and good levels of interconnected porosity. Further magnetic characterisation and induction heating investigations of these materials were performed.

### 2.3. Ca_2_Fe_2_O_5_ Magnetic Microspheres (Mass Ratio 1:1; Gas Flow Setting 2:2)

#### 2.3.1. Magnetic Properties of Ca_2_Fe_2_O_5_ Microspheres

**Figure 7:**
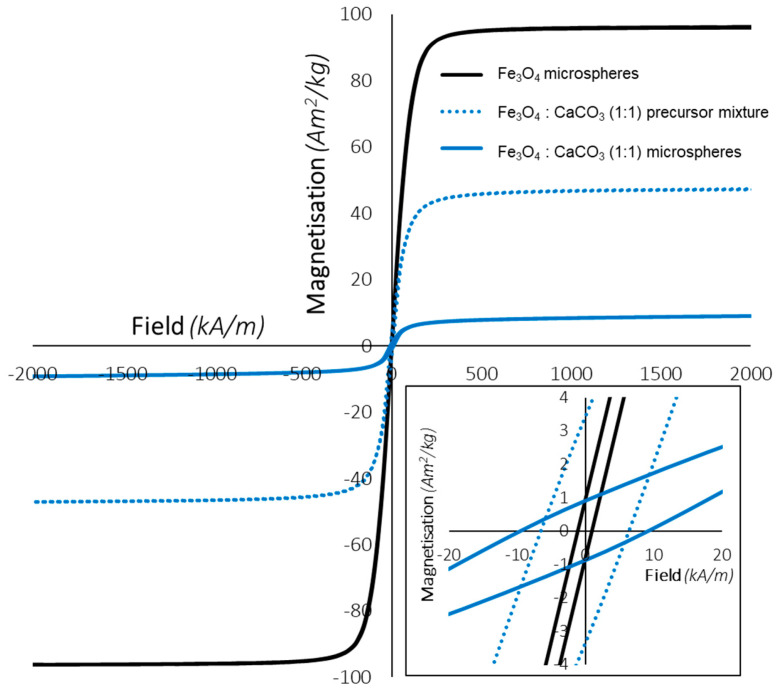
Hysteresis loop measurements for flame-spheroidised Fe_3_O_4_ microspheres, compared to Fe_3_O_4_:CaCO_3_ (mass ratio 1:1) precursor powders and microsphere products, at 26.85 °C. Inset figure provides evidence of remanent magnetisation in all cases.

Figure 7 presents magnetisation curves for the precursor/porogen mixture 1:1 Fe_3_O_4_:CaCO_3_, prior and post flame spheroidisation, compared to Fe_3_O_4_ dense microspheres processed in isolation, i.e., without CaCO_3_ porogen. As summarised in Table 6, the incorporation of porogen into the mixture led to a decrease of magnetisation saturation values. Nevertheless, it was noted that Fe_3_O_4_:CaCO_3_ flame-spheroidised products still showed significant magnetic saturation values (8.9 Am^2^/kg).

#### 2.3.2. Induction Heating Studies

**Figure 8:**
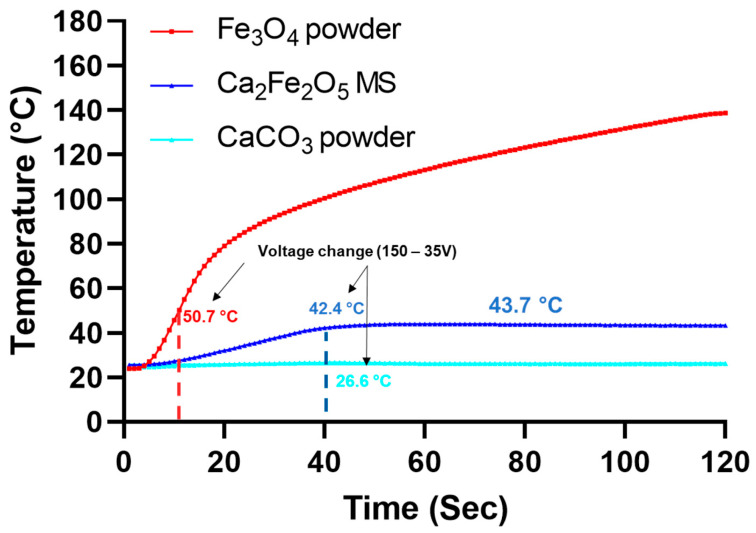
Induction heating curves for flame spheroidised Ca_2_Fe_2_O_5_ microspheres (MS), compared with Fe_3_O_4_ and CaCO_3_ starting powders. Homogenous Ca_2_Fe_2_O_5_ microsphere exhibited highly stabilised temperature control. (All curves display averages of triplicate measurements) (Statistical analysis on Supplementary Materials, Table S4).

The potential of Ca_2_Fe_2_O_5_ microspheres for magnetic-mediated hyperthermia was evaluated via induction heating (Table 7). Figure 8 shows the evolution of temperature for the most homogeneous flame-spheroidised Ca_2_Fe_2_O_5_ sample (mass ratio 1:1, gas flow setting 2:2), along with Fe_3_O_4_ and CaCO_3_ starting powders by way of control. The Fe_3_O_4_ powder showed high levels of induction heating, up to ~130 °C, but with an evident lack of heating control, whilst CaCO_3_ powder showed no induction heating as anticipated. Notably, homogeneous Ca_2_Fe_2_O_5_ microspheres exhibited highly controlled heating to a constant level of 43.7 °C which remained stable upon voltage decrease (150 to 35 V).

## 3. Discussion

This study reports on the optimisation of the flame-spheroidisation process parameters for the controllable production of magnetite-based porous microspheres (Fe_3_O_4_ precursor powders/CaCO_3_ porogen mass ratio, and gas flow setting). Modification of these parameters produced a variety of products in terms of shape (dense and porous microspheres, and irregular-shaped particles), or a mixture of these with distinct magnetic saturation levels and compositions (Fe-Ca excess/deficit). Optimised parameter conditions were identified for the manufacture of compositionally uniform and porous products, with Fe_3_O_4_:CaCO_3_ (mass ratio 1:1; gas flow setting 2:2) mixtures producing Ca_2_Fe_2_O_5_ microspheres with strong levels of compositional homogeneity and porosity levels (for the case of large porous microspheres). The homogeneous Ca_2_Fe_2_O_5_ samples demonstrated controlled delivery of heat (43.7 °C, see Figure 8), highlighting the suitability of these candidate products for magnetic hyperthermia applications.

**Figure 9:**
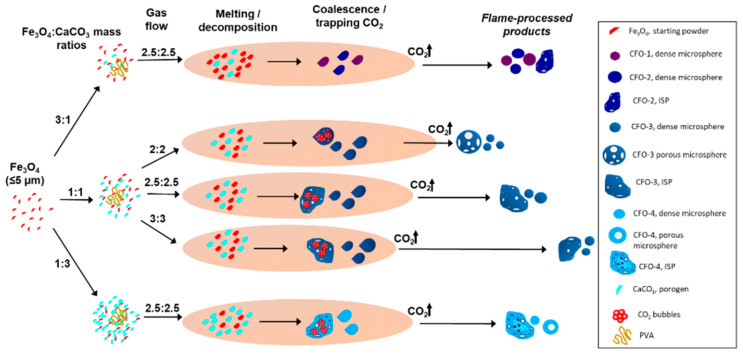
Flame spheroidisation of Fe_3_O_4_:CaCO_3_ microsphere products, as a function of mass ratio and gas flow settings.

Figure 9 provides a schematic illustration detailing the development of magnetic microspheres, as a function of mass ratio and gas flow setting parameters. Mass ratio parameters as applied to magnetite/porogen combinations revealed a direct effect on microsphere composition and magnetic properties. Importantly, Ca_2_Fe_2_O_5_ (srebrodolskite) was the only calcium iron oxide phase revealed for all Fe_3_O_4_:CaCO_3_ flame-spheroidised samples (Figure 2). The suggestion is that rapid cooling and solidification mechanisms associated with the flame-spheroidisation process allowed for the formation of Ca_2_Fe_2_O_5_ microsphere structures with modified compositions (excess/deficit of Fe/Ca atoms), i.e., the microsphere products retained structural integrity for all mass ratio cases but presented Fe/Ca variations according to elemental availability. This could also be attributed to the unusual capacity of Ca_2_Fe_2_O_5_ to support a number of defects [44,45]. Indeed, this phenomenon was reinforced by compositional analyses of sieved samples. MLA mappings (Figure 4) highlighted a clear trend towards iron deficit/calcium excess, as the mass ratio progressed from 3:1 towards 1:3, with a mass ratio of 1:1 showing the highest levels of homogeneity (CFO-3, denoted as Ca_2_Fe_2_O_5_). This compositional trend emphasised the importance of maximum consumption of the starting materials occurring for a mass ratio of 1:1 and gas flow setting of 2.5:2.5; in which case MLA data showed no evidence for any unreacted Fe_3_O_4_ and CaCO_3_ from this sample. It should be noted that sieving acted simply to improve sample homogeneity by removing excess, small, unreacted Fe_3_O_4_ and CaCO_3_, and Fe_2_O_3_ reacted powders. In contrast, Fe-rich samples (mass ratio 3:1) showed poor compositional uniformity, with two banded calcium iron oxide minerals observed (CFO-1 and CFO-2). Similarly, Ca-rich samples (mass ratio 1:3) were associated with low porogen consumption, along with irregular-shaped particles containing an excess of unreacted CaCO_3_ and reacted CaO. In addition, the mass ratio parameter also strongly influenced the microsphere magnetic properties. An increase in porogen content (from Fe_3_O_4_:CaCO_3_ 3:1 to 1:3) was directly related to a decrease in magnetic saturation and remanent magnetisation. This was attributed to the incorporation of paramagnetic calcium atoms within the srebrodolskite structure (Ca_2_Fe_2_O_5_), as a function of mass ratio. In this context, a report [46] is noted on the incorporation of Gd^3+^ within nanocrystalline iron oxide particles produced by an extraction pyrolytic technique, with hysteresis loops measured via vibrating sample magnetometry (VSM; magnetic field max. 795.7 kA/m) as a function of concentration (mol%) and temperature (at much slower heating rates compared to the rapid flame spheroidisation process). An increase in Gd^3+^ content (from 12.5 to 75 mol%) was related directly to a decrease in magnetic saturation values and remanent magnetisation, similar to the case of CaCO_3_. Notably, the flame spheroidisation process leads to the formation of metastable products with modified ferromagnetic properties.

The parameter of the gas flow setting also influenced the development of porosity within the microspheres. Two types of porosity were identified for this sample set: i.e., internal pores (either interconnected or not) and surface pores. It is considered that surface pores formed via molten droplets trapping and releasing CO_2_ gas bubbles produced during porogen decomposition (CaCO_3_ → CaO + CO_2_). In contrast, internal pores were created within molten drops as a consequence of unreleased CO_2_ gas bubbles, during rapid solidification. Indeed, it is noted that an increment in porogen concentration combined with an elevated gas flow setting, i.e., 3:3, was associated with higher internal porosity levels. Conversely, surface pores (with interconnected porosity) were more strongly associated with increased porogen content, albeit with a 2:2 gas flow setting. This effect was attributed to the increased residence time of molten droplets within the oxy-acetylene flame, as a determining factor for the development of microsphere porosity. Considering that particle temperature is directly related to the residence time of the particle within the flame [47,48], a gas flow setting of 2:2 would facilitate CO_2_ trapping and release, and maximise the number of reacted precursor/porogen powders, thereby producing fewer irregular-shaped particles. Furthermore, flame length could be controlled by adjusting the gas flow ratio [49]. As illustrated in Figure 9, the flame length decreased with increasing gas flow settings (from 2:2 to 3:3), consequently influencing particle residence time within the flame and cooling rate. In addition, polyvinyl alcohol (PVA) promoted the binding of Fe_3_O_4_ precursors with CaCO_3_ porogen particles, by helping to hold the agglomerated masses together. Accordingly, it is suggested that porous microspheres were produced from the agglomeration of Fe_3_O_4_:CaCO_3_ particles, with rapid melting and coalesce leading to the production of melt pools rendered spherical by surface tension, in advance of rapid solidification and phase separation, as appropriate.

Induction heating measurements demonstrated the capability of Ca_2_Fe_2_O_5_ microspheres (mass ratio 1:1; gas flow setting 2:2) to deliver heat in a controllable way, addressing one of the main limitations of magnetic hyperthermia which is controlling the temperature increase to between 40–45 °C [50]. It is suggested that the mechanism of heat generation used in our study was hysteresis loss, as revealed by magnetisation curves showing remanence (Figure 7; Inset figure). This hysteresis loss mechanism is associated with multi-domain, ferro- and ferrimagnetic materials [28,34], different from Néel and Brownian relaxation, responsible for heat generation within single-domain, superparamagnetic nanoparticles (SMNPs). Importantly, the induction heating parameters used for Ca_2_Fe_2_O_5_ measurements were similar to that previously reported for ferromagnetic glass-ceramic microspheres (denoted P40-Fe_3_O_4_ microspheres) [51] with the distinction that induced magnetic fields were higher for P40-Fe_3_O_4_ products. Regarding the different magnetic saturation levels between Ca_2_Fe_2_O_5_ (8.9 Am^2^/kg) and P40-Fe_3_O_4_ (4 Am^2^/kg), there was a requirement to adjust the field in order to reach the target temperature (via induction coil heating). Moreover, magnetic hyperthermia effects may be achieved through the application of weak magnetic fields (<7.95 kA/m) [3]; hence, relatively low magnetic fields were used for these Ca_2_Fe_2_O_5_ microsphere induction heating studies. Furthermore, the field frequency (204 kHz) used was within the clinically accepted range for magnetic hyperthermia [3,30,50,51,52,53,54,55]. Additionally, in the present induction coil experiments the target temperature was achieved rapidly (~40 s), indicating that these microsphere products are promising candidates for reduced periods of magnetic hyperthermia exposure, thereby preventing and reducing patient discomfort [3]. These induction heating measurements showed promising results for the Ca_2_Fe_2_O_5_ magnetic microspheres developed. However, for formal validation, this part of the investigation requires further study using alternating magnetic fields, similar to those found in clinical settings.

The formation mechanisms associated with Ca_2_Fe_2_O_5_ porous and dense microspheres developed via the flame spheroidisation process have been established [42]. For these magnetic microspheres, Fe_3_O_4_:CaCO_3_ particles were fed into a high-temperature oxy-acetylene flame (~3100 °C) where rapid melting and coalescence occurred. The molten particles acquired a spherical shape post exiting the flame due to surface tension. The development of compositionally uniform, porous and dense Ca_2_Fe_2_O_5_ microspheres, upon rapid cooling and solidification, was consistent with EDS data (Supplementary Materials, Figure S5 and Table S3) and CaO:Fe_2_O_3_ (2:1 molar ratio) of the Ca-Fe-O phase diagram [56]. Additionally, fine scale diffraction patterns (SAED data, Supplementary Materials, Figure S8) acquired from Ca_2_Fe_2_O_5_ microsphere fragments, confirmed their polycrystalline structure.

Accordingly, to produce porous microspheres with interconnected porosity and high values of compositional homogeneity, the results suggest that optimised flame spheroidisation process conditions should be at the gas flow setting of 2:2, using a magnetite-to-porogen mass ratio of 1:1. It was noted that the formation of compositionally uniform, Ca_2_Fe_2_O_5_ porous and dense microsphere products can be achieved by controlling the mass ratio and gas flow setting parameters from the flame spheroidisation process. Moreover, these Ca_2_Fe_2_O_5_ microspheres show potential for magnetic hyperthermia applications due to their ability to deliver heat in a controllable way. Additionally, the elevated temperatures of magnetic hyperthermia may improve synergistically the release of certain chemotherapeutic agents, such as cisplatin, cyclophosphamide and bleomycin [57,58,59]. Hence, the compositionally uniform Ca_2_Fe_2_O_5_ microspheres could be explored for drug delivery applications in combination with magnetic hyperthermia.

## 4. Materials and Methods

### 4.1. Materials 

The starting feedstock comprised mixtures of as-supplied iron(II,III) oxide powder (Fe_3_O_4_; ≤5 µm, 95%; Merck, Gillingham, UK) and calcium carbonate as porogen (CaCO_3_, ≤5 µm, 98%; Fisher Scientific UK Ltd, Loughborough, UK.). The magnetite (Fe_3_O_4_) powder was mixed with CaCO_3_ using a pestle and mortar and combined with droplets of 2% aqueous solution polyvinyl alcohol (PVA; Merck, UK) to act as a binder, followed by drying at 37 °C for 24 h.

### 4.2. Flame Spheroidisation Parameters

**Figure 10:**
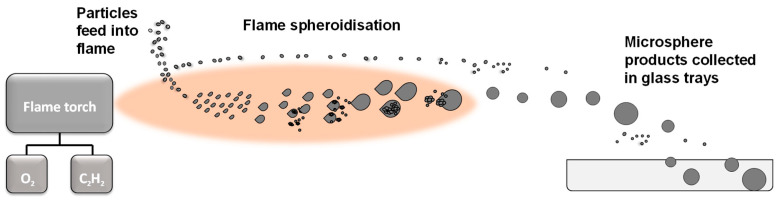
Schematic representation of the single-stage, flame spheroidisation process.

Fe_3_O_4_-based microspheres were produced by flame spheroidisation, whereby prepared powders were flame processed using a thermal spray gun (MK74, Metallisation Ltd., Dudley, UK) (Figure 10) [42].

The processed materials were collected using glass trays, placed a short distance away from the thermal spray gun, and stored in glass vials for characterisation. Parameters to evaluate the effects of Fe_3_O_4_ precursor to CaCO_3_ porogen mass ratios (3:1, 1:1 or 1:3) and oxy-acetylene gas flow settings (O_2_/C_2_H_2_; 2:2, 2.5:2.5 or 3:3) on the resultant products are summarised in Table 8 and Table 9, respectively.

### 4.3. Microsphere Characterisation

#### 4.3.1. Microsphere Morphology and Size

Imaging of the as-acquired flame spheroidised products (unsieved) was performed by scanning electron microscopy (SEM; FEI XL30; 5 kV; spot size 2.5; 13.3 mm working distance, secondary electron (SE) imaging mode). Microsphere size distributions were established using ImageJ 1.51h software (National Institutes of Health, Bethesda, MD, USA). 

#### 4.3.2. X-ray Diffractometry

Structural characterisation was performed by X-ray diffractometry (XRD; Bruker D8 Advance, Da Vinci design with LYNXEYE XE-T detector in 1D mode; Cu Ka radiation (λ = 0.15406 nm); 40 kV and 40 mA; step size 0.02°; total time/step 29.8 s per datapoint; 21 °C).

#### 4.3.3. Magnetic Characterisation

The microsphere products were then sieved (using a stainless-steel frame; 203 × 50 mm; ≥32 µm mesh; VWR International) to filter out surplus starting material. Complementary magnetisation measurements were performed using a superconducting quantum interference device magnetometer (SQUID; Quantum Design MPMS-3 system; VSM mode; vibration amplitude 1.5 mm; 26.9 °C).

#### 4.3.4. Compositional Characterisation

The sieved microspheres were embedded in a cold epoxy resin and sectioned by sequential mechanical grinding (using 400, 800 and 1200 SiC grit papers) and polishing (6 and 1 µm diamond paste). The polished samples were then cleaned using deionised water and industrial methylated spirit (IMS) and dried before carbon coating. Backscattered electron (BSE) imaging and chemical analyses of sieved and sectioned microspheres were performed via SEM-based mineral liberation analysis (MLA), using an FEI Quanta600 MLA (20 kV; spot size 7) equipped with energy dispersive X-ray spectroscopy (EDS) for compositional analysis and associated Bruker/JKTech/FEI data acquisition software for automated mineralogy. 

#### 4.3.5. High Frequency Induction Heating

Induction heating studies of sieved microspheres were performed via high frequency induction (Cheltenham Induction Heating Ltd.; 35–150 V; 20–120 W; 0.6–0.8 A; 204 kHz). Glass vials containing the magnetic microspheres were placed at the centre of a water-cooled copper coil generating an alternating magnetic field, whilst the temperature was measured using a fibre optic temperature sensor (Neoptix Reflex Signal Conditioner). Control samples of starting Fe_3_O_4_ and CaCO_3_ powders were also investigated. All measurements were repeated three times.

## 5. Conclusions

Compositionally uniform, ferromagnetic Ca_2_Fe_2_O_5_ porous and dense microspheres have been developed via the rapid, single-stage, flame spheroidisation process using feedstock powder Fe_3_O_4_:CaCO_3_ combinations. Morphological, structural and compositional investigations provided evidence of the effect of Fe_3_O_4_:CaCO_3_ mass ratio, and O_2_/C_2_H_2_ gas flow setting parameters. Complementary SQUID magnetometry confirmed the ferromagnetic properties of flame-spheroidised products. The potential use of Ca_2_Fe_2_O_5_ microspheres (1:1 mass ratio/2:2 gas flow setting) for magnetic hyperthermia applications with a simple, but significant, induction heating measurement (43.7 °C) was shown. The combination of compositional control, high levels of porosity and functional properties (i.e., magnetic and thermal) achieved opens up new opportunities, to explore the application of magnetic microspheres for a range of biomedical challenges.

## Figures and Tables

**Table 1:** Size range of flam-spheroidised reaction products (dense microspheres, irregular-shaped particles (ISP), and microspheres with surface porosity), as a function of precursor to porogen mass ratio.

Mass Ratio Fe_3_O_4_:CaCO_3_	Gas Flow Setting/arb. Unit	Dense Microspheres/µm	ISP/ µm	Microspheres with Surface Porosity/µm
3:1	2.5:2.5	35–165	75–475	No
1:1		65–210	205–465	
1:3		40–145	40–195	

**Table 2:** Magnetic measurements of flame-spheroidised products as a function of precursor to porogen mass ratio.

Mass Ratio Fe_3_O_4_:CaCO_3_	Gas Flow Setting	Magnetic Saturation/M_s_	Remanent Magnetisation/M_r_	Coercive Field/ H_c_	
	Arb. Units	Am^2^/kg (emu/g)	Am^2^/kg (emu/g)	(kA/m)	(Oe)
3:1	2.5:2.5	36	3.3	9.1	115
1:1		10.1	0.6	7.0	87.5
1:3		3.6	0.3	7.2	90

**Table 3:** Mineral proportion (wt%) of flame spheroidised products, as a function of precursor to porogen mass ratio.

Mass Ratio Fe_3_O_4_:CaCO_3_	Gas Flow Setting/arb. Units	Fe_3_O_4_/wt%	Fe_3_O_4_ with Ca/wt%	CFO-1/wt%	CFO-2/wt%	CFO-3 Ca_2_Fe_2_O_5_/wt%	CFO-4/wt%	CaCO_3_/wt%	Particles Analysed via MLA
3:1	2.5:2.5	0.3	3.9	52.7	39.7	3.0	0.2	0.1	9544
1:1		0.1	0.3	0.1	1.2	96.5	1.8	0.0	4064
1:3		1.0	0.7	0.7	1.2	13.9	64.4	18.1	23,851

**Table 4:** Size range of flame spheroidised reaction products (dense microspheres, irregular-shaped particles (ISP), and microspheres with surface porosity), as a function of gas flow setting.

Mass Ratio Fe_3_O_4_:CaCO_3_	Gas Flow Setting /arb. Unit	Dense Microspheres/µm	ISP/ µm	Microspheres with Surface Porosity/µm
1:1	2:2	35–80	15–105	125–180
	2.5:2.5	65–210	205–465	No
	3:3	55–150	175–210	No

**Table 5:** Mineral proportion (wt%) of microsphere products as a function of gas flow setting.

Fe_3_O_4_:CaCO_3_ Mass Ratio	Gas Flow Setting	Fe_3_O_4_/wt%	Fe_3_O_4_ with Ca/wt%	CFO-1/wt%	CFO-2/wt%	CFO-3/wt%	CFO-4/wt%	CaCO_3_/wt%	Particles Analysed via MLA
1:1	2:2	0.0	0.0	0.0	0.1	99.6	0.1	0.1	1560
	2.5	0.1	0.3	0.1	1.2	96.5	1.8	0.0	4064
	3:3	0.1	0.1	0.4	2.1	94.1	3.0	0.1	4127

**Table 6:** Magnetic saturation, remanent magnetisation and coercive field values of Fe_3_O_4_ dense microspheres, and Fe_3_O_4_:CaCO_3_ (mass ratio 1:1) mixtures before and after flame-spheroidisation.

Sample	Magnetic Saturation	Remanent Magnetization	Coercive Field/Hc	
	Am^2^/kg (emu/g)	Am^2^/kg (emu/g)	(kA/m)	(Oe)
Fe_3_O_4_ dense microspheres	96.3	0.8	0.5	6.7
Fe_3_O_4_:CaCO_3_ (1:1) starting powders	47.0	3.6	6.7	84.9
Fe_3_O_4_:CaCO_3_ (1:1) microspheres	8.9	0.9	8.7	109

**Table 7:** Experimental parameters for induction heating investigations.

Voltage/ V	Power/ W	Current/ A	Magnetic Field/ kA/m (Oe)	Frequency/ kHz
150	120	0.8	0.10 (1.2)	204
35	20	0.6	0.07 (0.9)	204

**Table 8:** Parameters to evaluate the effects of Fe_3_O_4_:CaCO_3_ mass ratio.

Fe_3_O_4_ Size/µm	CaCO_3_ Size/µm	Mass Ratio Fe_3_O_4_:CaCO_3_	Preparation	Oxygen-Acetylene Gas Flow Setting/arb. Unit
≤5	≤5	3:1	Mixed with PVA	2.5:2.5
		1:1		
		1:3		

**Table 9:** Parameters to evaluate the effects of gas flow setting.

Fe_3_O_4_ Size/µm	CaCO_3_ Size/µm	Mass Ratio Fe_3_O_4_:CaCO_3_	Preparation	Oxygen-Acetylene Gas Flow Setting/arb. Unit
≤5	≤5	1:1	Mixed with PVA	2:2
				2.5:2.5
				3:3

## Data Availability

The raw data that support the findings of this investigation is available from the corresponding author upon reasonable request.

## Supplementary Materials

The following supporting information can be downloaded at: https://www.mdpi.com/article/10.3390/molecules28062523/s1, Table S1: Generalised summary of unsieved flame spheroidised product proportions, as a function of precursor to porogen mass ratio. FS: flame spheroidised; Table S2. Molar constituents (weight percentage) and mineral composition reference; Figure S1. MLA mineral colour code as a function of excess (or deficit) Fe or Ca concentration; Figure S2. Full MLA compositional analysis and modal minerology of flame spheroidised Fe_3_O_4_:CaCO_3_ (mass ratio 1:1; gas flow setting 2.5:2.5), following sieving and sectioning, demonstrating high levels of CFO-3-Ca_2_Fe_2_O_5_; Figure S3. Full MLA compositional analysis and modal minerology of flame spheroidised Fe_3_O_4_:CaCO_3_ (mass ratio 3:1; gas flow setting 2.5:2.5), following sieving and sectioning, demonstrating high levels of CFO-1 and CFO-2; Figure S4. Full MLA compositional analysis and modal minerology of flame spheroidised Fe_3_O_4_:CaCO_3_ (MR 1:3; GFS 2.5:2.5), following sieving and sectioning, demonstrating high levels of CFO-4; Figure S5. BSE images and elemental mappings showing iron, calcium and oxygen for flame spheroidised Fe_3_O_4_:CaCO_3_ (mass ratios 3:1, 1:1, 1:3), following sieving and sectioning; Table S3. EDS molar concentrations (wt%) and proposed equilibrium phases for flame spheroidised Fe_3_O_4_:CaCO_3_ as a function of mass ratio; Figure S6. Full MLA compositional analysis and modal minerology of flame spheroidisation Fe_3_O_4_:CaCO_3_ (mass ratio 1:1; gas flow setting 3:3), following sieving and sectioning, demonstrating high levels of CFO-3-Ca_2_Fe_2_O_5_; Figure S7. Full MLA compositional analysis and modal minerology of flame spheroidised Fe_3_O_4_:CaCO_3_ (mass ratio 1:1; gas flow setting 2:2), following sieving and sectioning, demonstrating high levels of CFO-3-Ca_2_Fe_2_O_5_; Table S4. Statistical analysis on induction heating experiments; Figure S8. Cumulative selected area electron diffraction (SAED) pattern for 120 stacked tilted fields of view, corresponding to compositionally uniform, flame spheroidised Fe_3_O_4_:CaCO_3_ (mass ratio1:1; gas flow setting 2:2), i.e. Ca_2_Fe_2_O_5_ microsphere fragments following grinding; Table comprising crystal planes and 2-Theta.
