# Supplementary material for: Optimisation of the Flame Spheroidisation Process for the Rapid Manufacture of Fe3O4-Based Porous and Dense Microspheres
Source: Molecules. 2023 Mar 9;28(6):2523. doi: 10.3390/molecules28062523 (PMC10053185; doi:10.3390/molecules28062523)
Supplement: Supplementary file 1 [file molecules-28-02523-s001.zip › molecules-2237199-supplementary.pdf]

molecules

MDPI

Supplementary Files

Optimisation of the Flame Spheroidisation Process for the Rapid Manufacture of Fe_3O_4-Based Porous and Dense Microspheres

Jesús Molinar-Díaz [1], John Luke Woodliffe [1], Elisabeth Steer [2], Nicola A. Morley [3], Paul D. Brown [1,2] and Ifty Ahmed [1,*]

[1] Advanced Materials Research Group, Faculty of Engineering, University of Nottingham, University Park, Nottingham NG7 2RD, UK
[2] Nanoscale and Microscale Research Centre, University of Nottingham, University Park, Nottingham NG7 2RD, UK
[3] Department of Materials Science and Engineering, University of Sheffield, Sheffield S1 3JD, UK
* Correspondence: ifty.ahmed@nottingham.ac.uk

Effect of Fe_3O_4:$CaCO_3$ mass ratio

Structural analysis

Table S1. Generalised summary of unsieved flame spheroidised product proportions, as a function of precursor to porogen mass ratio. FS: flame spheroidised

Mass ratio Fe_3O_4:$CaCO_3$	FS products *(strong / medium / weak)*				
	Fe_3O_4	Fe_2O_3	$Ca_2Fe_2O_5$	$CaCO_3$	CaO
3:1	m	s	m	w	-
1:1	m	s	s	s	-
1:3	w	m	s	m	s

Mineral composition reference and colour code

Table S2. Molar constituents (weight percentage) and mineral composition reference.

Mineral	Molar constituents (wt%)		Mineral reference (%)			
	CaO	Fe_2O_3	Ca	Fe	O	C
Fe_3O_4	N/A	N/A	0	72.4	27.6	0
Fe_3O_4 with Ca	12	88	3.6	73.8	22.6	0
CFO-1	28.5	71.5	9.6	67.3	23.1	0
CFO-2	40.7	59.3	15.1	61.3	23.6	0
CFO-3 – $Ca_2Fe_2O_5$	48.3	51.7	19.1	57	23.9	0
CFO-4	82.4	17.6	48.4	28.9	22.6	0
$CaCO_3$	N/A	N/A	40	0	48	12

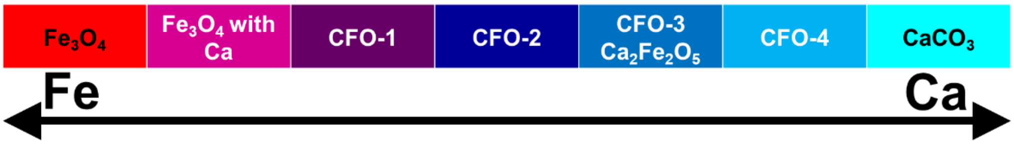

Figure S1. MLA mineral colour code as a function of excess (or deficit) Fe or Ca concentration.

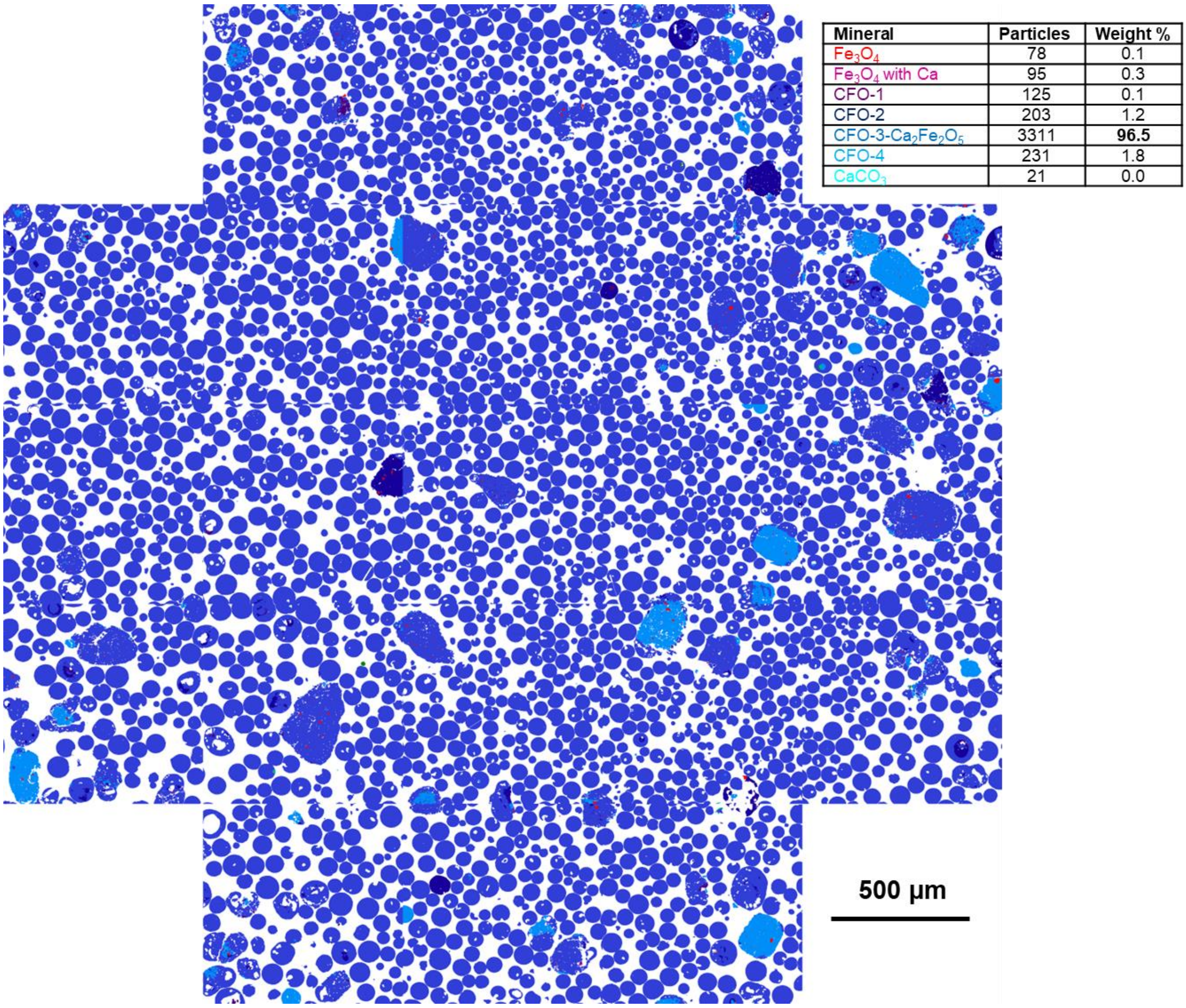

Mineral	Particles	Weight %
Fe_3O_4	78	0.1
Fe_3O_4 with Ca	95	0.3
CFO-1	125	0.1
CFO-2	203	1.2
CFO-3-$Ca_2Fe_2O_5$	3311	**96.5**
CFO-4	231	1.8
$CaCO_3$	21	0.0

Figure S2. Full MLA compositional analysis and modal minerology of flame spheroidised Fe_3O_4:$CaCO_3$ (mass ratio 1:1; gas flow setting 2.5:2.5), following sieving and sectioning, demonstrating high levels of CFO-3-$Ca_2Fe_2O_5$.

Mineral	Particles	Weight %
Fe_3O_4	573	0.3
Fe_3O_4 with Ca	2056	3.9
CFO-1	3250	**52.7**
CFO-2	2030	**39.7**
CFO-3-$Ca_2Fe_2O_5$	1199	3.0
CFO-4	278	0.2
$CaCO_3$	158	0.1

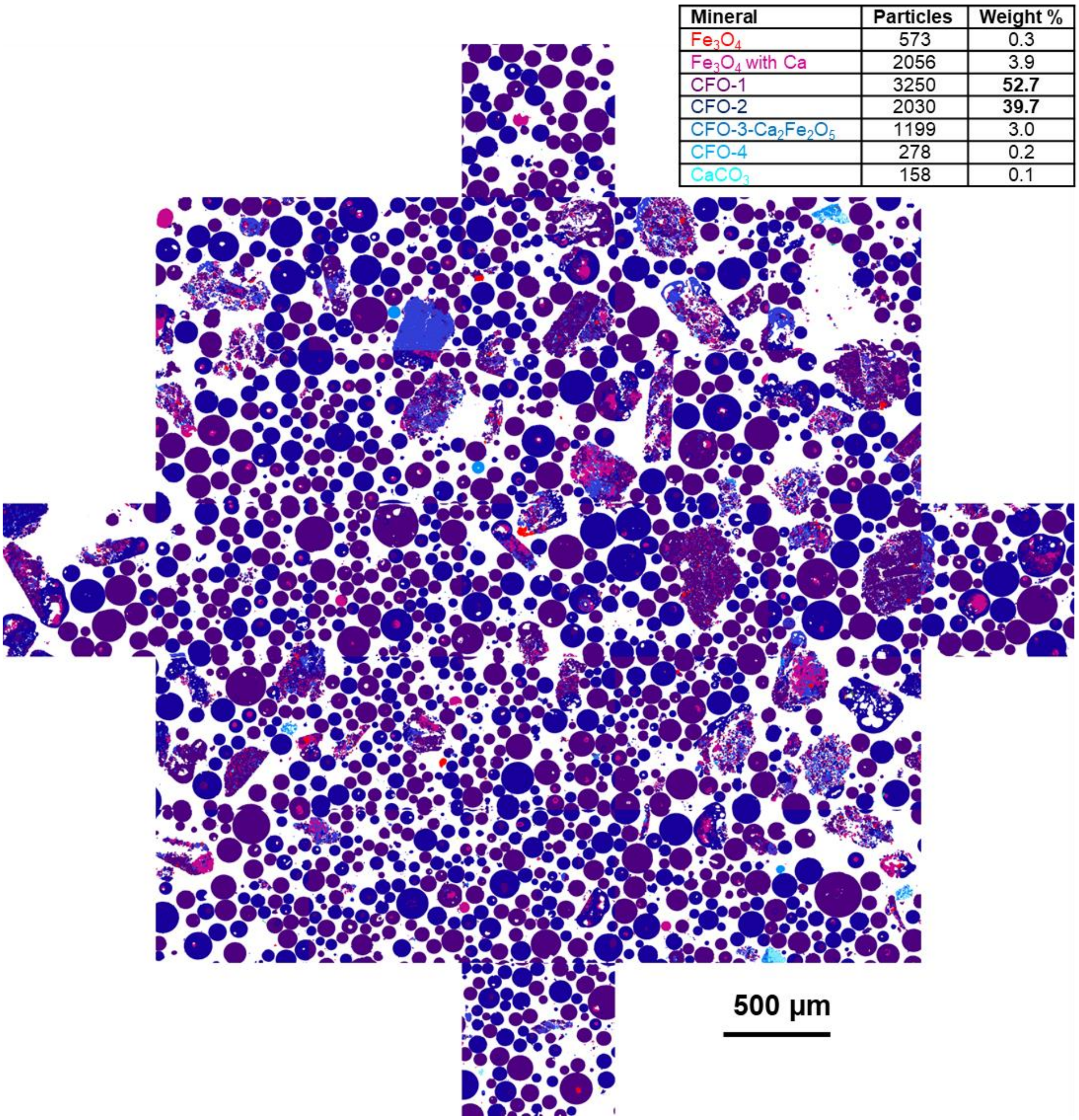

Figure S3. Full MLA compositional analysis and modal minerology of flame spheroidised Fe_3O_4:$CaCO_3$ (mass ratio 3:1; gas flow setting 2.5:2.5), following sieving and sectioning, demonstrating high levels of CFO-1 and CFO-2.

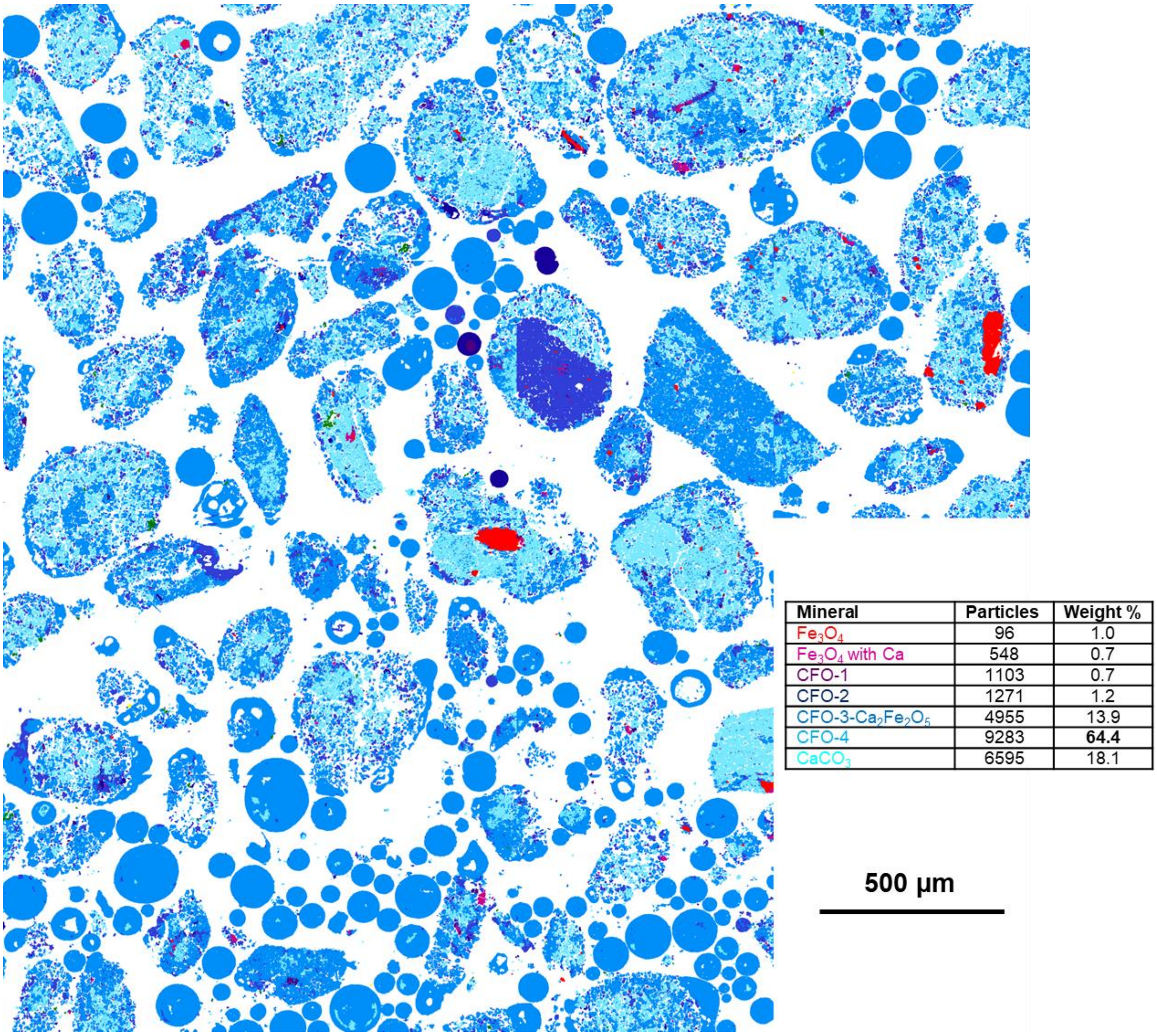

Mineral	Particles	Weight %
Fe_3O_4	96	1.0
Fe_3O_4 with Ca	548	0.7
CFO-1	1103	0.7
CFO-2	1271	1.2
CFO-3-$Ca_2Fe_2O_5$	4955	13.9
CFO-4	9283	**64.4**
$CaCO_3$	6595	18.1

Figure S4. Full MLA compositional analysis and modal minerology of flame spheroidised Fe_3O_4:$CaCO_3$ (MR 1:3; GFS 2.5:2.5), following sieving and sectioning, demonstrating high levels of CFO-4.

Energy dispersive X-ray (EDS)

Complementary energy dispersive X-ray (EDX; BRUKER software, FEI Quanta 600; 20 kV; 12.9 mm working distance) analysis investigations were performed to appraise elemental distributions across sectioned Fe_3O_4:$CaCO_3$ (3:1, 1:1, 1:3) microspheres. Figure S5 presents BSE and elemental maps of the microsphere products, as a function of mass ratio. Table S3 highlights the molar concentrations of decomposed porogen, *i.e.*, CaO; and oxidised magnetite, Fe_2O_3; associated with the production of $Ca_2Fe_2O_5$, following 2CaO + $Fe_2O_3 \rightarrow Ca_2Fe_2O_5$, in advance of droplet solidification.

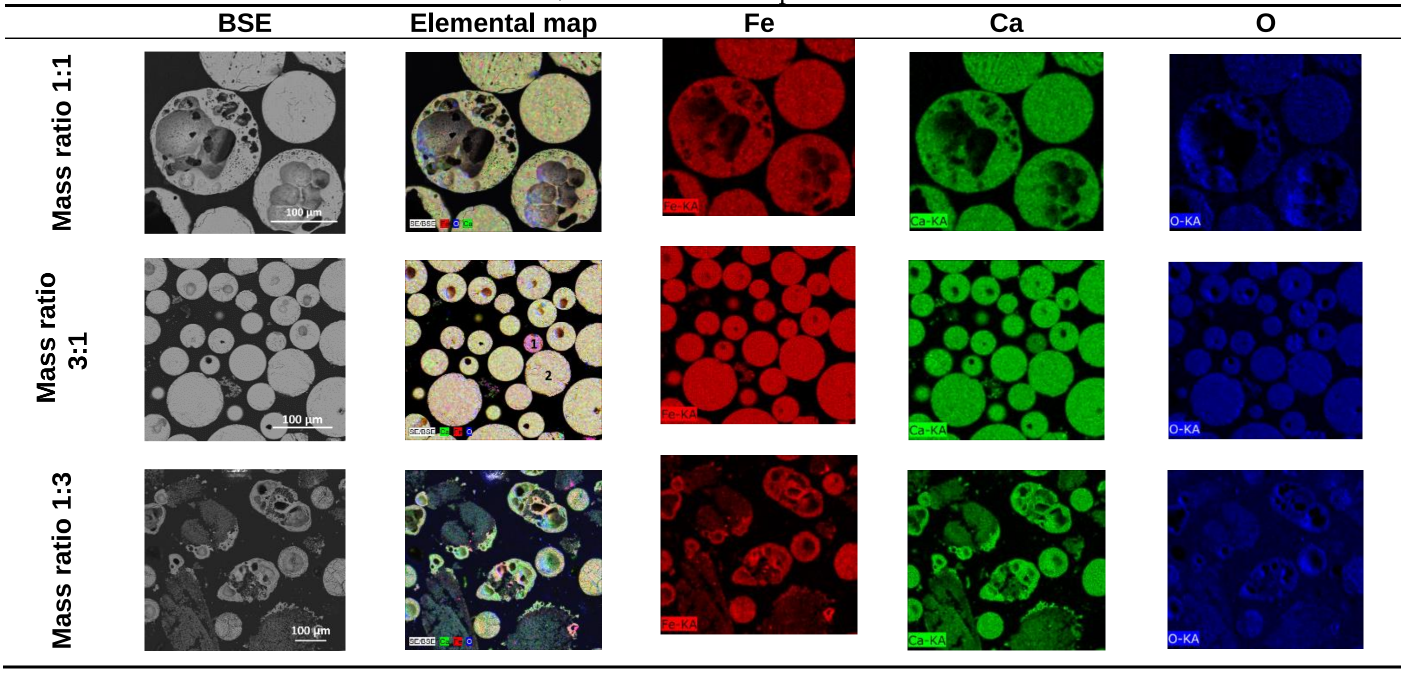

Figure S5. BSE images and elemental mappings showing iron, calcium and oxygen for flame spheroidised Fe_3O_4:$CaCO_3$ (mass ratios 3:1, 1:1, 1:3), following sieving and sectioning.

Table S3. EDS molar concentrations (wt%) and proposed equilibrium phases for flame spheroidised Fe_3O_4:$CaCO_3$ as a function of mass ratio.

Fe_3O_4:$CaCO_3$	CaO wt%	Fe_2O_3 wt%	Proposed phases
Mass ratio 1:1	66.06 ± 1.84	33.93 ± 1.84	CaO + $\mathbf{Ca_2Fe_2O_5}$
Mass ratio 3:1	29.57 ± 2.10	70.42 ± 2.10	$\mathbf{Ca_2Fe_2O_5}$ + $CaFe_2O_4$
	42.71 ± 1.54	57.28 ± 1.54	CaO + $\mathbf{Ca_2Fe_2O_5}$
Mass ratio 1:3	77.34 ± 5.62	22.65 ± 5.62	CaO + $\mathbf{Ca_2Fe_2O_5}$

Effect of gas flow setting

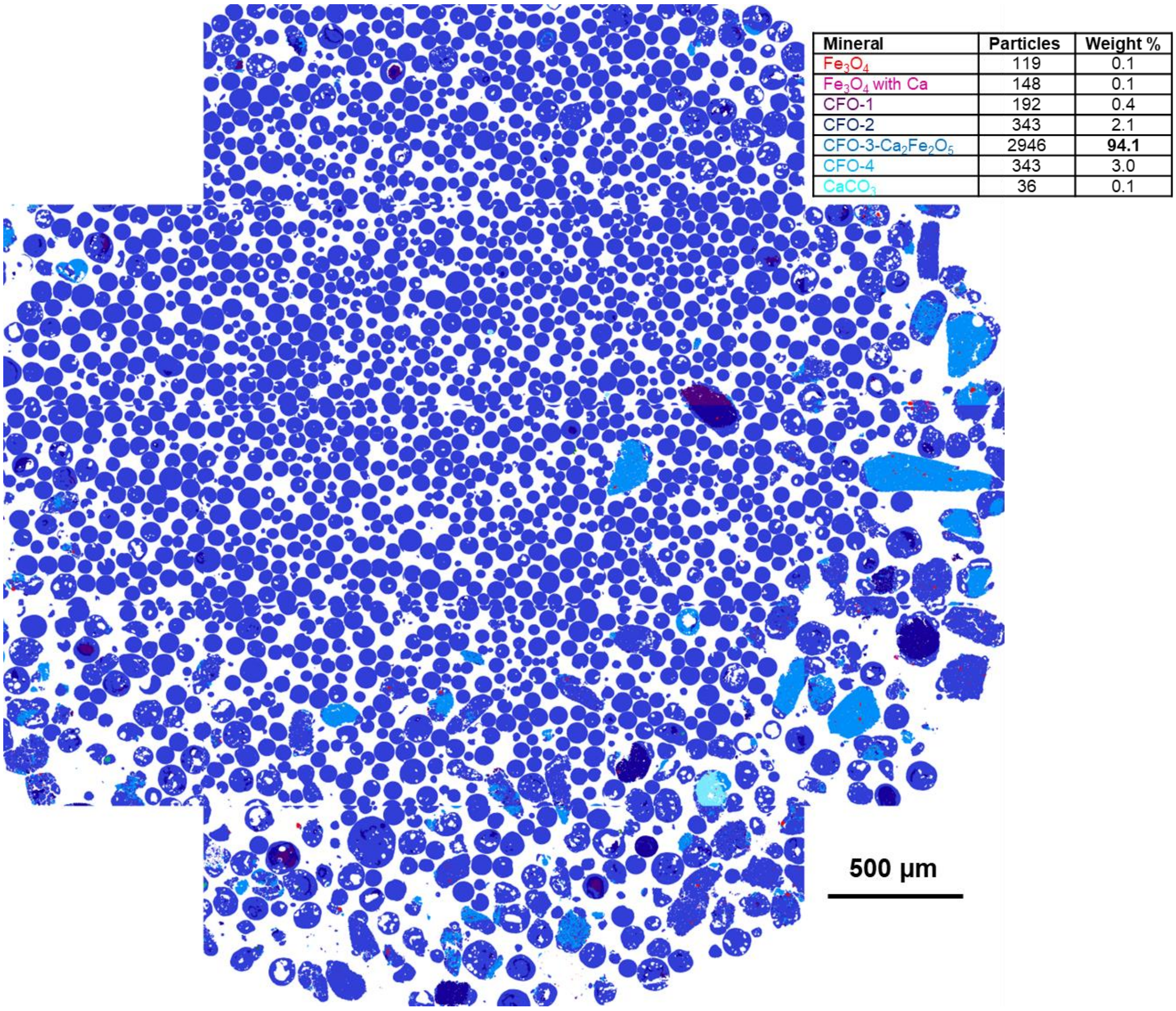

Mineral	Particles	Weight %
Fe_3O_4	119	0.1
Fe_3O_4 with Ca	148	0.1
CFO-1	192	0.4
CFO-2	343	2.1
CFO-3-$Ca_2Fe_2O_5$	2946	**94.1**
CFO-4	343	3.0
$CaCO_3$	36	0.1

Figure S6. Full MLA compositional analysis and modal minerology of flame spheroidisation Fe_3O_4:$CaCO_3$ (mass ratio 1:1; gas flow setting 3:3), following sieving and sectioning, demonstrating high levels of CFO-3-$Ca_2Fe_2O_5$.

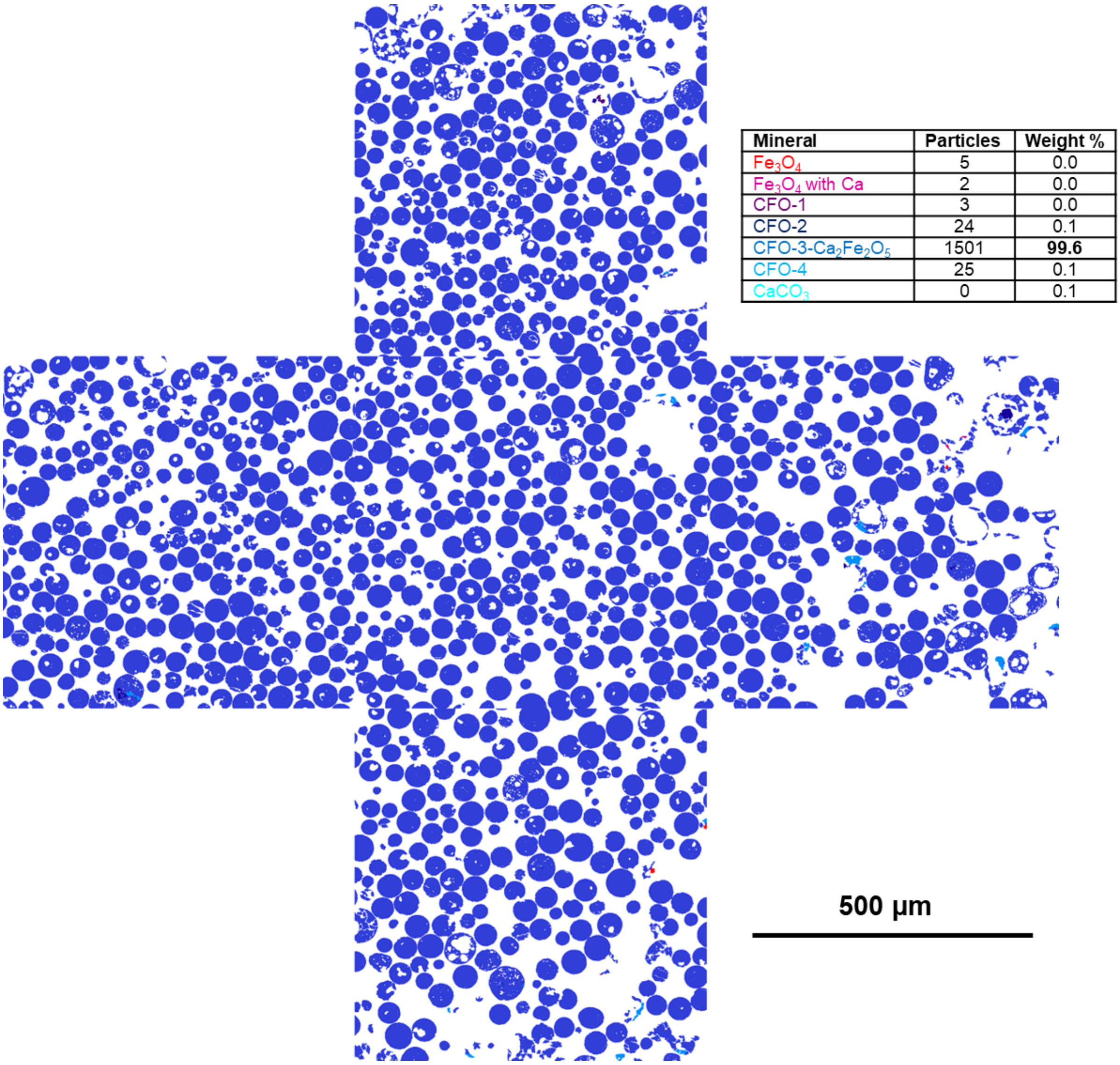

Figure S7. Full MLA compositional analysis and modal minerology of flame spheroidised Fe_3O_4:$CaCO_3$ (mass ratio 1:1; gas flow setting 2:2), following sieving and sectioning, demonstrating high levels of CFO-3-$Ca_2Fe_2O_5$.

$Ca_2Fe_2O_5$ microspheres (mass ratio 1:1; gas flow setting 2:2)

Induction heating – Std. errors

Table S4. Statistical analysis on induction heating experiments.

Std. Error	$Ca_2Fe_2O_5$ microspheres	Fe_3O_4 powder	$CaCO_3$ porogen
Slope	0.006150	0.02827	0.0009041
Y-intercept	0.4288	1.971	0.06303

Diffraction contrast patterns

Compositionally uniform, flame spheroidised Fe_3O_4:$CaCO_3$ (MR-1:1; GFS 2:2), dominated by $Ca_2Fe_2O_5$ MS, was ground using a mortar and pestle, dispersed in suspension and deposited on to an amorphous holey carbon film on a Cu TEM support grid. Diffraction contrast imaging was performed using transmission electron microscopy (TEM) (JEOL 2100+ TEM; 200 kV; operational mode selected area electron diffraction (SAED)) for automated acquisition series (range of -40 to +40 degrees tilt). Stacked 120 tilted diffraction patterns were loaded and analysed using Crystallographic Tool Box (CrysTBox) software.

Figure S8 presents a cumulative selected area electron diffraction (SAED) pattern from fine-scale, $Ca_2Fe_2O_5$ microsphere fragments. Well defined rings are evident, indicative of a fine-grained polycrystalline material. Diffraction ring identification matched crystal planes - 0 2 0, 2 0 0, and 1 4 1- corresponding to $Ca_2Fe_2O_5$ crystal structure.

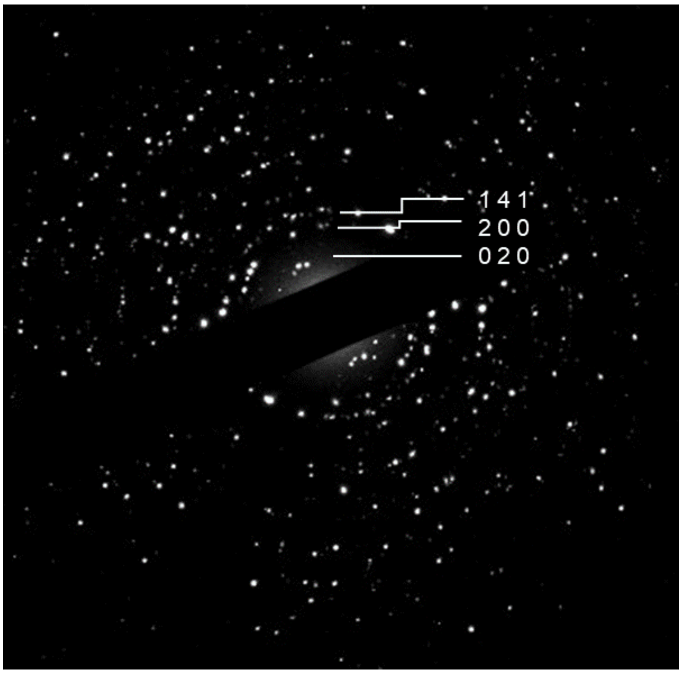

2-Theta	HKL
11.98	020
32.97	200
33.40	141

Figure S8. Cumulative selected area electron diffraction (SAED) pattern for 120 stacked tilted fields of view, corresponding to compositionally uniform, flame spheroidised Fe_3O_4:$CaCO_3$ (mass ratio1:1; gas flow setting 2:2), i.e. $Ca_2Fe_2O_5$ microsphere fragments following grinding; Table comprising crystal planes and 2-Theta.
